# Supplementary material for: Netcombin: An algorithm for constructing optimal phylogenetic network from rooted triplets
Source: PLoS One. 2020 Sep 18;15(9):e0227842. doi: 10.1371/journal.pone.0227842 (PMC7500971; doi:10.1371/journal.pone.0227842)
Supplement: S4 File — (ZIP) [file pone.0227842.s004.zip › SequenceData-Triplet/sequenceData-10-20-30-0-Triplet/length 400, numseq 40, rate 10^-7/5 seq.pdf]

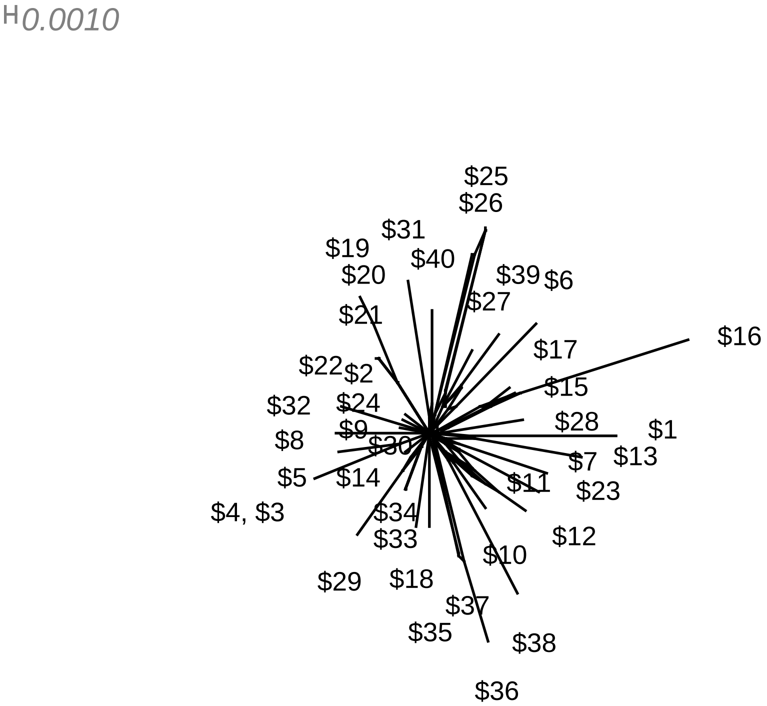

0.0010
$25
$26
$31
$19
$40
$20
$39
$6
$27
$21
$16
$17
$22
$2
$15
$32
$24
$28
$9
$1
$8
$30
$13
$7
$5
$14
$11
$23
$4, $3
$34
$33
$12
$10
$29
$18
$37
$35
$38
$36
